# Supplementary material for: Are carbon emissions trading and green financial instruments synergistic? -Comprehensive quantitative research based on content analysis
Source: PLoS One. 2024 Mar 7;19(3):e0298601. doi: 10.1371/journal.pone.0298601 (PMC10919601; doi:10.1371/journal.pone.0298601)
Supplement: S1 Data — (ZIP) [file pone.0298601.s001.zip › Data/地级市Z_F工作报告.zip!/╜¡╦╒/─╧╛⌐╩╨/└┤╫╘╣1⁄2╓┌║┼í╛┬φ┐╦╩2╛▌═°í┐_2015.docx]

各位代表：

现在，我代表市人民政府，向大会报告工作，请予审议，并请政协委员和其他列席人员提出意见。

2014年工作回顾

刚刚过去的2014年，是南京发展进程中很不寻常、很不平凡的一年，大事多、要事多、喜事多。一年来，面对复杂多变的国内外宏观环境和困难压力交织并存的特殊挑战，在市委正确领导下，我们全面贯彻党中央国务院和省委省政府的决策部署，紧紧团结和依靠全市人民，围绕打赢“经济发展稳中向好、改革创新实质突破、青奥盛会精彩圆满”三场硬仗，统筹推进稳增长、促改革、调结构、重生态、惠民生、防风险各项工作，较好地完成了市十五届人大二次会议确定的年度目标任务。

一年来，我们把办好青奥会和保障服务国家公祭仪式作为重中之重、头等大事，坚决按照党中央国务院和省委省政府部署安排，举全市之力，集各方之智，扎扎实实做好城市运行、综合保障、现场组织等各项工作，推动两件大事永载史册。青奥会精彩圆满，赢得了海内外高度评价，兑现了向国际社会作出的郑重承诺，实现了习近平总书记提出的“办得精彩、办出中国特色、体现国家形象”目标，向世界传播了“中国好声音”，为奥林匹克事业发展积累了“南京经验”，提升了南京的知名度、美誉度和国际影响力。国家公祭活动顺利举办，实现了“正正规规搞公祭”目标，展现了南京开放自信、和平博爱的国际形象，展示了南京人民不忘历史、珍爱和平、开创未来的坚强意志。

一年来，我们牢牢把握稳中求进、改革创新核心要求，坚持把经济工作重心放到提高质量和效益上，把经济增长动力转到依靠科技创新、深化改革上，攻坚克难、锐意进取，经济社会发展呈现出稳中向好、转中提质的良好态势。

——综合实力迈上新台阶。预计全年实现地区生产总值8800亿元，增长10%，增速高于全省和苏南平均水平；一般公共预算收入903.5亿元，同口径增长11%；全社会用电量增长1.7%。在15个副省级城市中，我市地区生产总值、服务业增加值增幅均居第1位；一般公共预算收入、规模以上工业增加值等实现了进位；各项贷款和存款新增量均居第3位，比上年末分别上升1位和2位。

——结构调整取得新成效。预计高新技术产业总产值占规模以上工业总产值比重达44%，提高2.4个百分点；六类九大战略性新兴产业主营业务收入增长15.5%，其中，新能源汽车、卫星应用、轨道交通等6个产业增幅超过20%；服务业增加值增长11.4%，其中，软件和信息服务产业主营业务收入增长27%，旅游业总收入首次突破1500亿元。

——综合改革激发新活力。承接行政审批事项88项、取消行政审批事项109项、下放行政审批事项104项；703项非行政许可审批不再保留，成为继天津之后全国第二座没有非许可审批城市；实施了工商登记制度改革，推行网上“多证联办”，出台鼓励民营经济发展“1+5”政策文件，全年新增企业4.36万户、增长50.6%，新增企业注册资金2545亿元、增长81.8%。

——城市品质实现新提升。如期完成青奥重点工程建设，国际青年文化中心、青奥村、“南京眼”步行桥成为南京城市新地标；着力加强生态环境建设，滨江风光带、金牛湖成为市民观光休闲新景观；大力实施环境综合整治，机场高速、绕城公路成为整洁靓丽新通道；全面推进重大基础设施建设，轨道交通、快速化通道成为百姓出行新选择；精心做好展示策划，明城墙、夫子庙成为南京对外新名片。

——民计民生有了新改善。持续加大民生投入，城乡公共服务支出占一般公共预算支出比重达75%；预计城乡居民人均可支配收入分别增长9%、10.5%；分两大区域实施低保同标，城乡困难群体首次享受同等待遇；新开工保障性住房347万平方米，竣工414万平方米；在全省率先实行学前一年基本免费教育；医养融合养老服务模式与和谐社区建设全国领先。

过去一年，我们科学应对宏观形势，强化经济组织运行，经济保持平稳较快发展。面对经济下行压力，坚持把稳增长作为首要任务，加强形势研判，采取有力措施，确保经济稳定运行在合理区间。强化政策创新，坚决落实国务院和省稳增长促改革调结构惠民生各项政策措施，结合南京实际，加大政策创新和集成力度，市级层面共制定实施112个政策文件，全力支持科技创新、实体经济、环境保护和民生事业发展。投资、消费、出口协同拉动，把政府投资与社会投资有机结合，更加重视激发民间投资活力，吸引社会资本投入基础设施、重点产业、社会事业等领域；加强重大项目推进，对154个重大项目实施挂图作战，对中电熊猫G108液晶面板等10亿元以上项目实施重点跟踪，增强了经济发展后劲。全力扩大消费需求，着力培育信息服务、网络购物等新型业态，加快发展文化、教育、体育、养老、健康等消费服务，社会消费品零售总额增长13%。制定并落实扶持外贸稳定增长一揽子政策措施，完成进出口总额572亿美元、增长2.7%；服务外包离岸执行额增长33.6%。

过去一年，我们坚持转型升级导向，推动结构战略调整，经济质量效益显著提升。坚持以科技创新引领产业转型，在结构调整优化中促进经济提质增效。创新驱动战略深入实施，更加注重加强知识产权保护，实施《南京市紫金科技人才创业特别社区条例》，实行科技创新券制度，提升创业载体孵化功能，促进产学研协同创新，搭建公共服务平台51个，科技创业特别社区新建载体201万平方米，集聚“321计划”人才1381名；发明专利申请量增长35%，每万人有效发明专利拥有量超过25件、居全国同类城市前列。产业结构明显优化，战略性新兴产业投资占全市工业总投资比重提高2个百分点，建材、冶金和石化等高能耗行业投资下降23%；服务业增加值占地区生产总值比重达55.8%，提高1.4个百分点；新增设施农业6万亩，新增家庭农场364家，休闲农业综合收入达46亿元。成功创建“全国质量强市示范城市”。节能减排全面完成，建立区域排污权交易机制，实施四大片区工业布局调整，完成20家中小工业企业搬迁关停；完成131家“三高两低”企业整治，预计万元GDP能耗下降7%，煤炭消耗下降3%。

过去一年，我们扎实推进改革开放，不断创新体制机制，发展动力活力明显增强。扎实推进第二轮综合改革，7大类48项改革任务全部完成方案制定，已审议出台30项。在行政审批制度改革不断深化、工商登记制度改革全面实施同时，国有资产管理改革实现突破，组建东南集团和扬子集团，国有资本从传统工商业领域退出24家企业，完成14家企业混合所有制改革。公共资源交易制度改革进展顺利，成立公共资源交易管理委员会，组建市公共资源交易中心，整合建设工程、政府采购、产权交易和土地招拍挂等7类交易活动，公共资源交易纳入规范化、法制化轨道。完成市级功能区体制改革，实施“市属区管”新体制，激发了基层的积极性和创造性。强化街道公共服务和社会服务职能，四城区街道全部取消经济指标考核，将更多精力投入到社会管理服务之中。

实施户籍制度一体化改革，市域范围内实现户口通迁。金融、财税、土地、社保、科技、教育、医疗卫生、资源性价格、社会治理等改革正有序推进。与此同时，加强与上海自贸区全方位对接互动，南京综合保税区开始实质性运作，龙潭跨境电子商务产业园等正式运行；两岸产业协同发展和创新试验区获国家批准。外事、对台、港澳、侨务等工作同步推进。

过去一年，我们优化城乡空间布局，加强基础设施建设，区域城市化展开新篇章。制定区域城市化规划，组织实施新区建设等九大工程，全面推进城市化和城市现代化。新区新城建设步伐加快，河西新城南部地区开发建设全面启动，青奥博物馆等青奥配套工程全面建成，金融城一期、博览中心二期等重点项目如期推进，展现了现代化国际性城市形象；江北新区前期工作有序开展，空港新城、溧水新城、高淳新城和10个新市镇加快建设，城乡区域空间布局进一步优化。基础设施建设实现重大进展，7条轨道交通线路同步建设，地铁10号线、宁高城际、宁天城际一期等3条线路建成通车，全市轨道交通通车里程由85公里提高到180公里，位列全国第4；禄口机场二期建成使用，机场高速扩建、江东路快速化改造、城西干道桥改隧、江北大道建设等工程全面建成，宁高新通道、纬三路过江隧道全力推进，火车站北广场、南京汽车客运新站投入使用。老城改造有序推进，下关滨江、铁北、燕子矶、铁心桥—西善桥等片区改造顺利推进，完成城中村、危旧房改造295.5万平方米。25公里明城墙本体修缮完毕，科举博物馆、六朝博物馆等一批文博场馆，相继建成并对外开放。农田水利、国土整治等工作取得明显成效。

过去一年，我们聚焦环境整治，狠抓综合治理，城市管理逐步走向长效常态。坚持问题导向，全城动员、全民发动，大力开展“大干一百天、环境大扫除”和“再干两百天、环境再提升、管理见长效”环境整治专项行动，着力解决人民群众反映强烈的突出问题。市容市貌焕然一新，完成22条主次干道、48条背街小巷环境综合整治，禄口机场、南京南站、玄武湖周边等窗口地区，以及进出城主要公路、铁路沿线环境全面提档升级；完成动迁项目110个、拆违1.38万处，拆违面积近170万平方米，拆除违规广告标识2.1万块，清运卫生死角垃圾50万吨。交通秩序大为改善，大力实施公交优先战略，新增和优化公交线路76条，新增、更新公交车2530辆，新增公共自行车7400辆；推行公交换乘优惠政策，主城区公交出行分担率达到46%；调整背街小巷停车管理体制，实施分区分级停车收费管理，新增停车泊位9.1万个，交通拥堵指数在全国排名从年初第6名下降到第21名。长效管理初步建立，出台城市长效管理意见，制定33类、2000个条目的城市综合治理指导标准；市本级城市维护费用由6亿元增加到10亿元，城管执法队员配比由万分之1.86增加到万分之2.71；城区道路机扫率达到80%，比上年提高20个百分点。

过去一年，我们强化大气污染防治，推进生态文明建设，生态环境质量持续改善。制定出台39项环境保护政策文件，严格落实相关治理体系和措施，生态环境不断优化，通过“国家生态市”考核验收。大气环境得到有效治理，大力实施50个大气减排重点项目，累计完成1012台燃煤锅炉的关停和清洁能源改造，南钢、金陵石化、扬子石化等一批脱硫脱硝工程全部建成投运；全面推行车用国Ⅴ汽柴油，淘汰黄标车6.1万辆；全方位治理在建工地扬尘，全密封管理3000辆渣土运输车，城区降尘量下降22%，全市PM2.5年平均浓度下降5.38%。水环境治理得到强化，集中整治河西南河、南十里长沟、滁河、水阳江等一批河道，完成玄武湖生态治理一期工程，铁北污水厂二期、城南污水厂二期等污水处理新扩建项目顺利推进。生态保护建设得到加强，在全市划定13类104块生态红线保护区域，对老山、金牛湖等一批生态资源实施重点保护；推进明外郭—秦淮新河风光带等重点工程建设，52公里滨江风光带一期工程、玄武湖东岸环境综合整治工程全面完成，青奥森林公园、鱼嘴湿地公园等一批生态工程顺利建成；建设美丽乡村示范区475平方公里，全市绿化造林6.2万亩。

过去一年，我们大力实施民生项目，加快发展社会事业，市民百姓满意度不断提升。扎实做好民生工作，民生十件实事全部完成。就业再就业工作扎实有效，新增城镇就业22.5万人，实现再就业10.4万人，城镇登记失业率为2.5%；完善鼓励支持大学毕业生就业创业优惠政策，新增城镇就业中大学以上人员占比提高4个百分点。社会保障水平再上台阶，城乡基本社会保险覆盖率达98%以上，被征地农民社会保障实现全覆盖，企业退休人员月人均基本养老金增长11%以上；城镇职工、居民医保和新农合政策范围内住院报销比例，分别提高到84%、74%和75%。建成市级居家养老综合服务中心、102个3A级社区居家养老中心，新增116个医护型居家养老服务中心，成为全国养老服务业综合改革试点城市。教育卫生事业均衡发展，开展中小学校长教师“区管校用”试点，扩大了优质教育资源覆盖面，成为第一批国家级义务教育均衡发展省辖市；河西儿童医院、市中医院新院、市公共卫生医疗中心等项目建设加快，城乡15分钟健康服务圈基本形成。文化体育事业全面进步，文化产业增加值增长15%，“文化建设工程”指标列全省第一；城市社区“10分钟体育健身圈”建设全覆盖，人均拥有公共体育设施面积提前一年实现现代化指标。社会治理不断创新，诚信体系建设扎实推进，安全生产、食品药品安全、信访维稳工作得到加强，平安南京、法治南京建设取得新成效，群众安全感达93.4%。双拥、民族、宗教、档案、史志、参事、社会科学工作取得新进展，计生、妇女、儿童、青少年、仲裁、残疾人、慈善事业取得新进步。

过去一年，我们着力转变政府职能，切实改进工作作风，政府自身建设得到加强。认真落实中央和省委市委有关规定，加强制度建设，密切联系群众，切实改进作风。决策更为科学，制定实施《南京市人民政府议事决策规则（试行）》等一系列政策文件，完善市政府常务会、市长办公会等议事决策机制，建立健全重大决策和重大项目民意征集、专家咨询，以及政府法律顾问、城乡规划管理等制度，政府决策科学化、民主化、法治化水平有了新提高。效能明显提升，扎实推进投资项目并联审批，审批时限控制在45天以内；充分发挥“12345”政务服务热线作用，全年办结率达99.8%，回访满意率达93.3%；在市级机关开展“公众开放日”活动；通过“省市政风行风热线联动直播走进南京”活动，与市民互动，限期督办了650条咨询、建议和投诉。作风得到加强，会议、创建达标、节庆论坛等活动大幅下降，规范性文件数量同比减少三分之一；市政府40个组成部门预算全部公开，“三公经费”支出比上年决算数下降11.3%。全年办理市人大代表议案8件、建议392件，办理市政协提案673件，办理质量不断提高；提请人大审议地方性法规草案6件，制定政府规章5件。

各位代表，过去一年，我们不辱使命、不负重托，较好完成了各项既定目标任务。成绩来之不易，经验弥足珍贵。这是市委贯彻党中央国务院和省委省政府决策部署，统揽全局、正确领导的结果，是全市人民团结拼搏、不懈奋斗的结果。在此，我代表市人民政府，向全市人民表示崇高敬意和衷心感谢！向各位人大代表、政协委员，向各民主党派、工商联、无党派人士和各人民团体、各界人士，向驻宁人民解放军、武警官兵和人民警察，向中央驻宁单位和省级机关，表示衷心感谢！向关心和支持南京发展的港澳台同胞、海外侨胞和国际友人，表示诚挚谢意！

在看到成绩的同时，我们也清醒地认识到，当前我市经济社会发展中还存在一些矛盾和薄弱环节。影响经济社会发展的体制机制问题尚未得到根本解决，综合改革工程仍需深入推进；内需不旺与外需不稳并存，经济增长动力不足、下行压力加大，部分企业生产经营困难，结构调整任重道远；土地节约集约利用水平不高，资源环境约束趋紧，节能减排压力较大，市民反映强烈的灰霾天气、河道污染、部分节点交通拥堵等问题仍然突出；城乡区域发展还不平衡，养老、教育、医疗等民生保障工作与群众期望还有差距，改善民生仍需加大力度；城乡居民收入持续增长难度加大，既定的增收目标未能实现；政府职能转变还不到位，依法行政亟需提高，一些工作人员为民服务、勤政廉政、尽责履职的意识和能力有待进一步加强。对此，我们一定高度重视，采取有力措施，认真加以解决。2015年总体要求和目标任务

今年是全面深化改革的关键之年，是全面推进依法治市的开局之年，也是全面完成“十二五”规划的收官之年。政府工作的总体要求是：全面贯彻党的十八大和十八届三中、四中全会，以及习近平总书记系列重要讲话精神，以总书记视察江苏和青奥会成功举办为强大精神动力，坚持稳中求进工作总基调，坚持以提高经济发展质量和效益为中心，主动适应经济发展新常态，坚定不移实施创新驱动战略，坚定不移转方式、调结构、稳增长、惠民生，坚定不移推进全面改革和依法治市，全力促进经济平稳健康发展和社会和谐稳定，确保“十二五”圆满收官，在“迈上新台阶、建设新江苏”的征程中干在实处、走在前列。

今年经济社会发展的预期性指标是：地区生产总值增长9.5%左右，一般公共预算收入同口径增长9%以上，固定资产投资增长9%以上，社会消费品零售总额增长13%左右，外贸出口保持正增长。约束性指标是：全体居民人均可支配收入增长与经济增长同步，农村居民人均可支配收入增速高于城镇居民人均可支配收入增速1个百分点以上。万元GDP能耗下降7%左右，煤炭消耗总量控制在3240万吨以内。结构性指标是：服务业增加值占地区生产总值比重提高1个百分点。民营经济增加值占地区生产总值比重提高2.5个百分点。全社会研发经费支出占地区生产总值比重达到3.5%。高新技术产业产值占规模以上工业产值比重提高1.5个百分点，科技创业投资增长15%。这些指标是市委市政府多方听取意见、慎重权衡后确定的，体现了党中央国务院和省委省政府有关精神，与我市“十二五”规划目标相衔接，反映了新常态下的增长可能、转型要求和就业需要。

做好今年工作，要全面落实习近平总书记视察江苏时的重要讲话精神，把总书记提出的“五个迈上新台阶”最新要求，作为建设新南京的根本遵循和强大动力，更加积极适应经济运行、城市建设管理、民生保障和改善三个新常态，以经济强夯实发展基础，以百姓富体现发展成效，以环境美增强发展优势，以社会文明程度高提升发展境界，坚定不移朝着新南京目标迈进，加快建设现代化国际性人文绿都。坚持转型发展，大力推进产业转型，加快发展战略性新兴产业、现代服务业和现代农业，加快构建现代产业体系；大力推进城市转型，切实更新城市规划理念，转变城市建设管理方式，着力提升城市现代化、国际化水平；大力推进政府转型，加快建设法治型、服务型政府。坚持创新发展，把科技创新、制度创新作为关键环节，通过实施创新驱动、改革攻坚，不断激发市场活力、增强内生动力、释放增长潜力、拓展发展空间，确保调速不减势、量增质更优。坚持协同发展，主动对接国家战略，处理好区域之间、城乡之间，以及发展与民生、经济与环境的关系，加快各种资源优化配置，推动各种力量优化整合，促进各种利益优化平衡。坚持和谐发展，紧紧围绕“更好的教育、更稳定的工作、更满意的收入、更可靠的社会保障、更高水平的医疗卫生服务、更舒适的居住条件、更优美的环境”的目标，解决好最广大人民群众的根本利益、部分群众的共同利益、特殊群众的特殊利益，筑牢和谐基石，确保大局稳定。

重点做好八个方面工作：

（一）深入推进综合改革，激发城市发展活力。全面贯彻中央和省委市委全面深化改革的决策部署，确保重点改革任务逐一落地、见到实效，努力在全面深化改革中走在前列。

释放民营经济活力。加快落实行政审批制度和工商登记制度改革等措施，切实实施好“五个文件、十张清单”，营造公平竞争的市场环境。落实好促进民营经济创新发展和混合所有制经济跨越发展系列政策文件，推出一批含金量高、收益预期好的重大项目，吸引社会资本进入基础设施、金融服务、市政工程、公共服务等领域。鼓励和引导民间资本参与混合所有制改造，重点推进50家市属国有企业改革改制和资产重组。组织银企对接活动，制定实施差别化金融支持政策，更大力度扶持小微企业发展。

激发创新创业活力。深化科技体制改革，通过创新券、风险补偿、后补助、创投引导等方式，按照市场规律引导支持企业技术创新活动。推动产学研深度融合，强化科技同经济的对接、创新成果同产业的对接、创新项目同现实生产力的对接、研发人员创新劳动同其利益收入的对接。深化人才管理体制改革，制定《南京市紫金科技人才创业特别社区条例》实施细则，优化完善“321”政策体系，加快创建紫金人才管理改革试验区，进一步推进大众创业、万众创新。

调动基层发展活力。加快构建“两级政府、三级管理、权责一致”政府管理体制，市级重点加强规划与国土管理、财政与公共政策、行政执法与监督等调控职能，区级重点加强经济发展和城市管理职能。落实好街道和社区改革措施，强化街道公共服务和社会服务职能。积极培育发展公益慈善、社区服务类社会组织，健全政府向社会组织购买公共服务机制。鼓励各区在城市治理、新型城镇化、国土空间优化再开发等方面大胆探索。深化区域金融、财税、土地使用制度、资源性产品价格等改革，加强诚信体系建设。

（二）坚持科技创新引领，加速产业转型升级。扎实推进创新驱动战略，以创新培育新的增长点，促进创新成果转化为高端产业、高端产品，加快形成新的发展动力源。

促进科技与产业对接。全面实施“一核两翼”策略，以紫金科技人才创业特别社区为核心，以高新区转型提升和东南科技创新带整合提升为两翼，加快形成创新驱动新局面。强化企业创新主体地位，推动江苏省产业技术研究院、未来网络创新研究院、北斗产业研究院等一批产业平台建设，加快形成一批可转化的成果，实现产业提质增效升级。新增战略性新兴产业创新中心8个、世界500强和中国500强企业研发机构20家、工程技术研究中心60家。

培育壮大新兴产业。以“六谷二十一园”为载体，大力发展未来网络、智能电网、生物医药等战略性新兴产业，确保软件和信息服务产业主营业务收入增长20%以上，六类九大战略性新兴产业主营业务收入增长15%以上，投资占工业投资比重提高3个百分点。加快推进比亚迪新能源汽车、中船海洋装备机电产业园、正大天晴创新药物研发生产基地、清华紫光集成电路产业园等重大项目，确保中电熊猫G108液晶面板等项目竣工投产。深入推进“两化融合”，提升传统产业竞争力。

加快发展现代服务业。坚持高端发展导向，大力发展移动电子商务、互联网金融、跨境电子商务等互联网经济，推动互联网与传统产业深度交融，创新商业模式和服务业态，支持苏宁易购、宏图三胞、焦点科技等企业发展壮大。加快发展现代物流、金融服务、会展旅游、商务商贸、文化创意、总部经济等现代服务业。大力推进服务业集聚区建设，开工建设金融城二期、国际博览中心三期，规划建设18个文化休闲旅游集聚区，以及健康服务、人力资源服务、知识产权、检验检测等产业园。启动南京华侨城综合文化旅游项目建设，加快推进世贸梦工厂动漫文化基地等重大项目。金融业增加值力争达到1000亿元，电子商务交易额、信息消费增长30%以上，服务外包离岸执行额增长25%。

全力以赴扩大内需。适应消费需求变化，以建设国家信息消费试点城市、电子商务示范城市、中国健康服务与生命科技名城等为契机，以“智慧南京”、“健康南京”、“畅游南京”为抓手，发展消费新业态，培育消费新热点，做大信息、文化、旅游、体育、养老等消费规模。着力优化投资结构，加大对科技创新、基础设施、生态环保、社会民生等领域的投入，全力以赴推进当年总投资超1300亿元的203个市级重大项目，促进投资合理增长。

（三）对接国家发展战略，打造新的经济增长极。抢抓“一带一路”、长江经济带等多重国家战略叠加机遇，在区域经济竞争合作中，打造新亮点、构建新优势。

启动江北新区建设。按照“规划先行、改革先行、立法先行、生态先行”原则，全面启动江北新区开发建设。进一步完善新区顶层设计，建立健全新区运行体制机制，加快制订《江北新区条例（草案）》。高标准做好空间规划、产业规划、城市设计等工作，为新区长远发展夯实基础。统筹做好新区项目谋划、招引和储备，建成六合机场，开展铁路南京北站前期工作，推进城市中心区建设、棚户区改造、生态环境建设等一批重大项目。

发展壮大枢纽经济。充分发挥南京发展航运物流独特优势和条件，优化港口布局，加快建设集疏运体系、物流园区和航运服务体系，加速发展航运交易、海事法律、航运经纪等服务业，推动南京与长江中上游城市港口联盟实质运行，加快打造长江区域性航运物流中心。强化航空枢纽功能，整合空港要素资源，推进空港综合保税区申报，重点发展航空物流、航空制造、临空关联产业，打造空港枢纽经济区。依托南京南站枢纽优势，大力发展商务商贸、科技研发、文化创意等产业，构建现代化智慧型高铁枢纽经济产业体系。

打造对外开放新平台。对接上海自贸区，加快南京综合保税区建设。抢抓苏南国家自主创新示范区建设机遇，支持高新区、江宁开发区等建设国家自主创新示范区。支持江宁高新园等创建国家级高新区，支持有条件的省级开发区申报国家级开发区。加快建设两岸产业协同发展和创新试验区。推进中新南京生态科技岛创建省经济和生态体制协同改革试验区。建成省级以上产业特色园区6家、生态工业园区9家、知识产权园区6家、创新型园区2家。

增创开放型经济新优势。创新对外开放思路和举措，扩大服务领域对外开放，提升利用外资质量。完成南京电子口岸一期建设，开展龙潭港启运港退税试点，推进禄口国际机场72小时过境免签，以及冰鲜水产品进口口岸建设。开展首批全国中医药服务贸易先行试点。加快跨境贸易电子商务产业园建设。推动外贸转型升级，提升外贸竞争力。积极参与“一带一路”建设，支持轨道交通、电力装备等优势行业和企业“走出去”。

（四）全面推进区域城市化，提升城市功能品质。转变城市建设管理方式，统筹建设重点，把握建设时序，构建“一新区、三新城、三枢纽、五片区”格局。

优化城市空间布局。以江北新区为重点加速区域城市化进程，加快河西新城现代化国际性城市新中心建设，科学有序推进溧水新城、高淳新城建设，加快建设空港、海港、高铁枢纽经济区。加快老城有机更新，整体改造提升下关滨江、铁北、燕子矶、铁心桥—西善桥、麒麟五个片区功能品质。严控主城区建设规模和开发强度，逐步疏散中心城区非核心功能。推进“多规融合”，开展城市开发边界划定试点，推进城镇低效用地再开发试点，健全土地统一储备运作机制。加强城市地下空间开发利用。

有序推进城市建设。按照“量力而行、适度超前”的原则，创新城建投融资机制，继续推进地铁4号线、宁和城际、宁高城际溧水段、宁高城际高淳段等轨道交通建设，开工建设地铁5号线和长江五桥、高淳至溧阳高速公路，建成纬三路过江隧道、宁高新通道，启动实施模范西路改造、纬七路东进、红山南路东延等一批城市道路工程。推进中新南京生态科技岛、东山副城、仙林副城等功能区基础设施建设，促进产城融合发展。推动紫金山—玄武湖生态文化旅游示范区建设，续建马群换乘中心。推进长江新济州河道治理工程，开工建设秦淮东河先导段和石臼湖、固城湖防洪能力提升工程。加快江南、江北环保产业园建设。

着力推进新型城镇化。编制新市镇、新社区和布点村建设规划，加大新市镇新社区建设力度，构建布局合理、功能错位的村镇体系。启动新一轮全域统筹工作，选择条件较好的若干镇街开展新一轮试点。加快交通水务一体化、社会保障一体化改革，稳步推进农民市民化。做好江宁街道、金牛湖街道全国发展改革试点城镇工作。健全南京都市圈协同发展六大机制，实施《宁镇扬同城化发展规划》。

健全城市长效管理机制。全力推进“再干两百天”环境综合整治，加大对市容市貌、道路街巷、停车秩序、渣土运输、户外广告、小区物业等重点领域管理，实现从突击整治向长效管理转变。强化区（园区）城市建设管理主体责任，推进城市管理养护市场化，建立完善“政府监管、建管分离、管养分开、市场竞争”城市管护新机制。推动全民参与城市治理，全面落实“门前三包”责任制。进一步集中城市管理领域行政处罚权，加大网格化巡防执法力度，完善城市管理考核机制，健全城市管理督查问责机制。提高城市管理精细化水平，实行24小时全时段覆盖的渣土运输管理，主城区主次干道机扫率达到85%以上。

（五）转变农业发展方式，扎实推动现代农业建设。坚持农业现代化与新型工业化、信息化、城镇化同步推进，加快转变农业发展方式，推动现代农业迈上新台阶。

深化农业农村综合改革。加快推进农村要素市场建设，力争镇街农村产权流转交易市场全覆盖，形成市、区、镇街三级联动的农村产权流转交易市场体系。全面推开土地承包经营权登记颁证工作。建立完善耕地保护长效机制，全力推进集体资产确权登记发证，加强村集体经济“三资”管理。扎实推进国家级农村改革试验区建设。

大力发展现代农业。全面启动整体创建国家现代农业示范区工作，把中国（南京）生物农业谷等“一谷四园”打造成全国一流的现代农业集聚区。加快推进农业科技进步，全面提高农业装备和技术水平。深化农业结构战略性调整，加快构建现代农业产业体系。推进休闲农业与乡村旅游融合发展，力争实现休闲农业综合收入48亿元。加强农业基础设施建设，新增高标准农田12万亩、设施农业4万亩，完成土地综合整治25万亩。

培育新型农业经营主体。加快培育专业大户、家庭农场、农民合作社、农业企业等新型经营主体，大力发展农机、农艺、植保等服务性合作组织，积极培养有文化、懂技术、会经营的新型农民队伍，力争新型经营主体农业适度规模经营面积占比超过50%，农民参加规范的专业合作社比重达69%，农业龙头企业销售收入增长10%。

加快推进美丽乡村建设。重点推进五大示范片区建设，确保新创建美丽乡村示范区300平方公里，新创建市级示范村30个以上，基本建成江宁、高淳美丽乡村示范区。

（六）加强生态文明建设，营造优美宜居环境。全面实施“保、调、减、退、提”生态环境倒逼策略，一手抓保护建设，一手抓污染治理，促进生态环境持续改善。

实行更严格的生态保护制度。大力实施区域排污权交易机制、废弃资源处理利用补偿机制、区域生态补偿机制、碳排放权交易市场建设等改革措施，大幅度提高排污费征收标准，以经济杠杆倒逼企业治污减排、转型升级。严格环境准入，明确全市禁止和限制建设的产业门类和空间区域，严格污染物排放总量控制、污染物排放标准和清洁生产等要求，形成产业结构、生态空间和总量控制“三位一体”的环境准入模式。加快推进四大片区工业布局调整，推动企业转型发展，启动烷基苯厂等4家重点骨干企业搬迁工作，完成9家中小企业搬迁关停。加快江南绕城公路以内工业生产企业“退城入园”步伐。全面推进全国绿色循环低碳交通运输区域试点城市建设。落实国家淘汰落后产能要求，化解过剩产能，淘汰落后产能。大力发展循环经济，建设国家资源综合利用“双百工程”示范基地。强化环境执法监管，从严打击各类环境违法行为，规范环境秩序，保障环境安全。

加强大气和水环境治理。加大灰霾整治力度，下大力气实施燃煤、工业、机动车、扬尘等污染协同治理，加强秸秆禁烧和综合利用，实施油烟污染专项整治，强化区域联防联控，参照青奥标准严格执行重污染天气预警和应急响应工作机制，确保全市PM2.5平均浓度较2013年下降7%，完成“十二五”减煤控煤任务。加强水环境治理，严格落实河长制，完成仙林污水处理厂二期扩建、高新区北部污水厂一期等工程，完成主城六区排水达标区建设267个。加强水资源保护，确保集中式饮用水源地水质达标率100%。积极开展土壤污染防治。加快推进垃圾分类工作，城区生活垃圾分类收集覆盖率达80%。

深化生态环境建设。巩固国家生态市创建成果，加快国家生态文明示范区建设。严格执行生态红线保护规划。实施“绿色南京”工程，确保完成绿化造林4万亩，林木覆盖率达到29.1%。继续推进滨江风光带二期等一批生态重点工程，加快建设老山、金牛湖等生态保护区。完成废弃露采矿山环境整治500万平方米，建成18个游园绿地，完成18条进出城主要道路绿色通道提升、15条国道省道和城乡道路绿色通道建设任务。

（七）促进文化繁荣发展，彰显城市特色形象。大力弘扬社会主义核心价值观，深化文明城市创建，推动文化事业繁荣和文化产业发展，建设独具魅力的人文都市。

大力发展文化事业。加强公共文化服务体系建设，继续推进文化惠民“百千万”行动计划，打响南京文化艺术节等一批文化品牌，启动南京演艺中心、南京艺术小学新校等项目建设。开展全民阅读活动，加快推进“书香南京”建设及“农家书屋”提升工程，实现全市公共图书馆“通借通还”。坚持以人民为中心的创作导向，多出优秀人才，多出精品力作。加强对外文化交流，举办首届南京国际青年节，办好米兰世博会“南京周”等活动。

加快发展文化产业。编制文化产业功能区布局和建设规划，发展新型文化业态，创建1家国家级文化产业园区、2—3家省级以上文化产业园区（基地）。推动文化与科技、金融深度融合，建设国家级文化和科技融合示范基地，争创“国家文化金融合作实验区”。建设南京创意设计中心、南京文化金融服务中心等公共服务平台，打造“南京攻略”文化旅游移动消费平台。做好文化企业上市融资工作，培育一批主业突出、实力雄厚的龙头企业，文化产业增加值占地区生产总值比重达6%以上。

保护传承历史文化。加强物质遗产和非物质遗产保护，完成明故宫遗址公园核心区、南朝石刻等重点区域保护规划，推进明城墙和海上丝绸之路·南京遗迹申遗工作，实施百子亭历史风貌区等一批近现代建筑保护和修缮。建成侵华日军南京大屠杀遇难同胞纪念馆三期工程，完成周边区域城市设计。加快明朝博物馆和城墙博物馆等建设，规划建设“云锦城”、石头城遗址公园等重点项目，完成梅园新村纪念馆改扩建工程，牛首山和大报恩寺遗址公园一期工程建成开放。

（八）坚持民生需求导向，持续增进人民福祉。围绕“七个更”的目标，统筹推进民生社会事业发展，不断提升公共服务水平，努力构建更加公平、普惠、均衡的民生保障新机制。

多措并举促进城乡居民增收。通过健全工资决定和正常增长机制、壮大股份合作经济、发展民营经济、鼓励创业就业和加强救助帮扶等措施，不断提高城乡居民收入水平。探索农民增收长效机制，更加关注低收入群体和困难群众，城乡居民收入达标人口比例提高4个百分点。

构建城乡一体社会保障体系。建立与经济发展水平相适应的社会保障待遇正常调整机制，完善养老服务和社会救助体系，加快城乡居民基本医疗保险制度并轨。加大住房保障力度，推进公共租赁住房与廉租房并轨运行，逐步建立以共有产权管理为核心的购买型住房保障制度。

推动公共服务均衡化发展。改善义务教育薄弱学校办学条件，全市域创成“江苏省学前教育改革发展示范区”。促进义务教育优质均衡发展、普通高中特色多样发展、职业教育双业提优发展。扩大教师“区管校用”试点范围。加快南京软件科技大学筹建工作。加快四大保障房片区学校配建，做好外来务工人员随迁子女入学工作。推进市中医院新院、浦口新城医疗中心等建设，确保市公共卫生医疗中心、河西儿童医院年底前投入运行。广泛开展全民健身活动，促进群众体育与竞技体育协同发展。提升双拥共建、优抚安置工作水平，争创“双拥模范城”八连冠。做好工青妇、民族宗教、援疆援藏、侨务、参事、史志、档案、地震、气象、社会科学等工作。

深化社会综合治理。加强基层社会管理服务体系建设，整合区、街（镇）、社区（村）社会服务资源，提高社会管理服务水平。推动社区减负增效常态化，加强和谐社区建设，全市98%的城市社区和96%的农村社区达到省和谐社区标准。继续改造提升老旧小区，创新物业管理机制，提高小区服务水平。落实安全生产责任制，加强安全生产监管，从源头消除安全生产隐患。强化食品药品安全保障，建立全程监管和责任追究制度。重视和加强信访稳定工作，更加注重运用法治方式预防和化解社会矛盾。深入推进平安南京、法治南京建设，完善立体化、现代化社会治安防控体系。

各位代表，人民群众对美好生活的向往，就是我们的奋斗目标。今年，市政府将重点办好民生十件实事。（1）提高保障水平。实现职工养老、失业、医疗、工伤、生育五险市级统筹，实行低保全城同标。启动城镇职工和居民大病保险。为全市户籍居民购买自然灾害公众责任保险、居民家庭财产综合保险。（2）完善住房保障。续建各类保障性住房950万平方米、新开工保障房280万平方米、竣工280万平方米。完成200万平方米的棚户区改造整治。（3）改善交通出行。新增、更新公交车1243辆，新改建公交场站27座，新增公共自行车1万辆以上，新增停车泊位6万个。（4）促进就业创业。新增城镇就业16万人，其中大学以上人员占比提高3个百分点，实现再就业6.5万人，援助困难人员就业1万人，转移农村劳动力4万人次，培育自主创业者1.1万人。（5）优化医疗健康服务。引导优质卫生医疗资源向新区、薄弱区布局，全市90%以上社区卫生服务中心、88%以上村卫生室达到省级建设标准。（6）推进教育均衡发展。托管学校98所，名校办分校30所，优质教育资源覆盖率达80%。新扩建达省优标准的幼儿园40所。（7）改善农村基本生产生活条件。新建改造农村公路400公里。完成农村特困户危房改造600户。人均纯收入低于6500元低收入农户全部脱贫。（8）加强养老服务。建成市3A级社区居家养老服务中心150个，新增养老机构床位4179张。（9）强化便民服务。制定实施《镇街便民服务中心公用服务事项基本目录》，整合便民服务功能，为市民提供优质便捷服务。新扩建社区综合服务中心30家以上。（10）推动智慧生活。基本实现全市公共区域WIFI网络免费向公众开放。以市民卡为载体，推进诚信卡试点。推广居民健康卡，实现区域医疗一卡通、一账通。

今年还有一项重要工作，就是编制“十三五”规划。我们将立足市情、发扬民主、凝聚智慧，编制一个适应时代要求、体现中央精神、符合南京实际、反映群众意愿的“十三五”规划，为南京新一轮发展奠定良好基础。建设人民满意政府

法治，是治国理政的基本方式；为民，是各级政府神圣职责。我们将全面贯彻依法治国基本方略，加快建设法治政府和人民满意的服务型政府。

严格依法行政。牢固树立法治意识、法治理念，坚持依法治市、依法执政、依法行政共同推进，法治城市、法治政府、法治社会一体建设，把依法行政落实到政府工作全过程，推动法治南京建设走在全国全省前列。研究制定法治政府建设实施规划，理顺行政管理体制，推进政府部门机构、职能、权限、程序、责任法定化，加快建设职能科学、权责法定、执法严明、公开公正、廉洁高效、守法诚信的法治政府。深化行政执法体制改革，规范行政执法行为，落实行政执法责任制，完善纠错问责机制，加大关系群众切身利益的重点领域行政执法力度。对行政机关重大合同一律严格实施合法性审查。建立重大行政决策报告备案制度、实施后评估制度、终身责任追究制度及责任倒查机制。

推进科学行政。正确处理好政府与市场的关系，做到以政府权力清单消除权力寻租，以负面清单释放市场活力，以责任清单规范政府行为。建立健全政府重大行政决策法定程序，严格遵守《关于加强“三重一大”事项决策和监管的意见》、《南京市重大行政决策程序规则》和《南京市人民政府议事决策规则》，推进政府决策和运行机制科学化、民主化、法治化。积极防范政府性债务等潜在风险，守住不发生系统性风险的底线。建立完善行政审批事项裁量基准制度、并联审批制度、监督管理制度，提升服务效能。推行公共服务标准化，加强电子政务建设，完善市政务服务中心及各级服务载体，构建综合高效的政务服务平台。

强化勤政廉政。深化“四风”整治，大力整治庸懒散拖等问题，坚决克服等待、观望和畏难情绪，保持勇于担当、甘于奉献、务实苦干的优良作风。强化宗旨意识，密切联系群众，有效打通联系服务群众的“最后一米”。厉行勤俭节约，反对铺张浪费，建设节约高效政府。对照“十二五”目标，着力补缺、补弱、补短和补软，实现“十二五”圆满收官。全面贯彻落实党风廉政建设责任制，强化源头治理，以零容忍态度惩治腐败，严肃查办发生在重点领域、关键环节和群众身边的腐败案件。稳步推进机关公务用车制度改革。进一步加强对财政资金分配使用、国有资产监管、政府投资项目管理、政府采购、公共资源转让、公共工程建设等重点领域和重点环节的规范管理，扎实推进惩治和预防腐败体系建设。全面实施政务公开，重点推进财政预决算、部门预决算、重大建设项目和社会公益事业信息公开，强化审计、监察、督查的监督作用，自觉置身于人大代表、政协委员、人民群众和社会舆论监督之下，确保权力在阳光下运行。

各位代表，“迈上新台阶，建设新南京”，责任重大，使命光荣。让我们紧密团结在以习近平同志为总书记的党中央周围，在中共南京市委的领导下，解放思想、克难奋进、扎实工作，为加快建设经济强、百姓富、环境美、社会文明程度高的新南京而不懈奋斗！
